# Sleeve gastrectomy improved microvascular phenotypes from obesity cohort, detected with optical coherence tomography angiography

**DOI:** 10.1111/1753-0407.13374

**Published:** 2023-03-05

**Authors:** Yaying Chen, Yanyang Liu, Lin Cong, Ailin Liu, Xiangyuan Song, Wenting Liu, Rong Hua, Qiwei Shen, Yikai Shao, Yiwen Xue, Qiyuan Yao, Yuyan Zhang

**Affiliations:** ^1^ Department of Ophthalmology Huadong Hospital, Fudan University Shanghai China; ^2^ Department of Ophthalmology Huashan Hospital, Fudan University Shanghai China; ^3^ Center for Obesity and Metabolic Surgery Huashan Hospital, Fudan University Shanghai China; ^4^ Department of Ultrasound Huashan Hospital, Fudan University Shanghai China

**Keywords:** bariatric surgery, metabolically healthy, metabolic syndrome, obesity, optical coherence tomography angiography, 减肥手术, 代谢健康型肥胖, 代谢综合征, 肥胖, 光学相干断层血管造影

## Abstract

**Aims:**

To examine how metabolic status is associated with microvascular phenotype and to identify variables associated with vascular remodeling after bariatric surgery, using noninvasive optical coherence tomography angiography (OCTA).

**Methods:**

The study included 136 obese subjects scheduled for bariatric surgery and 52 normal‐weight controls. Patients with obesity were divided into metabolically healthy obesity (MHO) and metabolic syndrome (MetS) groups according to the diagnosis criteria of the Chinese Diabetes Society. Retinal microvascular parameters were measured by OCTA, including superficial capillary plexus (SCP) and deep capillary plexus (DCP) vessel densities. Follow‐ups were performed at the baseline and 6 months after bariatric surgery.

**Results:**

Fovea SCP, average DCP, fovea DCP, parafovea DCP, and perifovea DCP vessel densities were significantly lower in the MetS group, compared to controls (19.91% vs. 22.49%, 51.60% vs. 54.20%, 36.64% vs. 39.14%, 56.24% vs. 57.65% and 52.59% vs. 55.58%, respectively, all *p* < .05). Parafovea SCP, average DCP, parafovea DCP, and perifovea DCP vessel densities significantly improved in patients with obesity 6 months after surgery, compared to baseline (54.21% vs. 52.97%, 54.43% vs. 50.95%, 58.29% vs. 55.54% and 55.76% vs. 51.82%, respectively, all *p* < .05). Multivariable analyses showed that baseline blood pressure and insulin were independent predictors of vessel density changes 6 months after surgery.

**Conclusions:**

Retinal microvascular impairment occurred mainly in MetS rather than MHO patients. Retinal microvascular phenotype improved 6 months after bariatric surgery and baseline blood pressure and insulin status may be key determinants. OCTA may be a reliable method to evaluate the microvascular complications associated with obesity.

## Graphical abstract

**Highlights**

- Different microvascular phenotypes were detected by noninvasive optical coherence tomography angiography (OCTA) in metabolically healthy obesity and metabolic syndrome patients.
- Baseline blood pressure and insulin status may be key determinants for vascular benefit from bariatric surgery.
- OCTA may be a reliable method to study the onset and progression of microvascular complications associated with obesity before and after bariatric surgery.

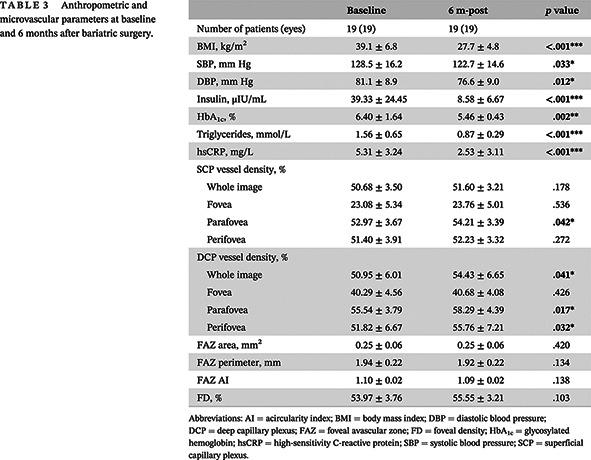

## 摘要

**目的:**通过非侵入性光学相干断层扫描血管成像(OCTA)检查, 分析代谢状态与微血管表型的相关性, 确定与减肥手术后血管重塑相关的变量。

**方法:**本研究包括136名计划行减肥手术的肥胖受试者和52名正常体重对照。根据中华医学会糖尿病学分会制定的诊断标准, 将肥胖患者分为代谢正常肥胖(MHO)组和代谢综合征(MetS)组。采用OCTA测量视网膜微血管参数, 包括浅层毛细血管丛(SCP)和深层毛细血管丛(DCP)血管密度。在基线和减重手术后6个月进行随访。

**结果:**与对照组相比, MetS组黄斑中心凹SCP、平均DCP、中心凹DCP、旁中心凹DCP和中心凹周围DCP血流密度均显著降低(分别为19.91%比22.49%, 51.60%比54.20%, 36.64%比39.14%, 56.24%比57.65%, 52.59%比55.58%, 均p < 0.05)。术后6个月, 肥胖患者中心凹旁SCP、平均DCP、中心凹旁DCP和中心凹周围DCP血流密度均较术前显著改善(分别为54.21%比52.97%, 54.43%比50.95%, 58.29%比55.54%, 55.76%比51.82%, 均p < 0.05)。多变量分析显示, 基线血压和胰岛素是术后6个月血管密度变化的独立预测因素。

**结论:**视网膜微血管损害主要发生在MetS患者, MHO患者较少。减肥手术后6个月视网膜微血管表型改善, 基线血压和胰岛素状态可能是关键决定因素。OCTA可能是评价肥胖相关微血管并发症的可靠方法。

## 1 INTRODUCTION

Obesity is a chronic metabolic disease that is characterized by excessive adipose tissue or abnormal adipose distribution, usually accompanied by weight gain.^1^ The prevalence of obesity has increased worldwide in the past 50 years, reaching pandemic levels.^2^ The deleterious metabolic effects of obesity are widely recognized at population level. Obesity confers serious health consequences such as coronary heart disease, type 2 diabetes, hypertension, cerebrovascular disease, and several kinds of cancers, thereby increasing all‐cause mortality.^3^, ^4^, ^5^ Interestingly, data from several studies suggest that mortality risk is increased in metabolically unhealthy obese individuals, but not metabolically healthy obesity (MHO), compared with normal‐weight individuals. Therefore, obesity can be further divided into MHO and metabolically unhealthy obesity, the latter is often called metabolic syndrome (MetS).^6^, ^7^ Body fat distribution, insulin resistance, lipid profiles, blood pressure, and inflammation are thought to be useful in distinguishing MHO from MetS.^7^ However, the underlying mechanism by which obesity increases the cardiometabolic disease risk, as well as the mechanism by which the two obese subgroups lead to different cardiovascular outcomes, remains unclear.

Microvascular dysfunction is an early manifestation of obesity‐related cardiometabolic disease and can ultimately lead to cardiovascular hemodynamic impairment.^8^, ^9^ There is a large body of retinal microvascular studies in relation to cardiometabolic risk factors and diseases.^10^, ^11^ Obesity is proved to be associated with narrower arteriolar and wider venular diameters, independent of other traditional cardiovascular risk factors.^12^, ^13^ Thus, the measurement of retinal microvascular function of obesity, including MHO and MetS, is important to help us to understand the pathophysiologic mechanisms that contribute to different outcomes of these two obesity subtypes.

Bariatric surgery improves different vascular biomarkers as pleiotropic effects of weight loss, reducing the risk of cardiovascular disease.^14^, ^15^, ^16^ Habib et al conducted a 24‐month follow‐up study on 50 patients with obesity and found that carotid intermedia thickness decreased and flow‐mediated brachial artery dilation increased in patients after bariatric surgery.^17^ A 36‐month postoperative follow‐up of 60 patients with obesity also showed significant improvements in aortic elasticity and left ventricular diastolic function.^18^ Nonetheless, the effect of bariatric surgery on microvascular structure and function remains unclear. Some studies have shown that coronary blood microcirculation in patients with obesity significantly improved in both the short term and long term after bariatric surgery.^19^, ^20^ However, other studies have found that bariatric surgery is associated with a paradoxical acute deterioration of microvascular impairment in patients with obesity with diabetic retinopathy.^21^ Further investigations are needed to determine the vascular impact of bariatric surgery on patients with obesity.

In the previous studies on retinal microvascular network of patients with obesity, traditional fundus photography and static retinal vascular analyzer were used to evaluate the retinal vascular diameter or arteriolar venular ratio,^12^, ^21^, ^22^ yet these static and manual measures could not be widely used in clinical practice. Recent progress in optical coherence tomography angiography (OCTA) has provided a more accurate technique that can quantitatively evaluate retinal and choroidal perfusion in vivo. Retinal and optic capillary plexus are visualized and segmented through a layer‐by‐layer analysis in the OCTA system.^23^, ^24^, ^25^ Retinal vessel density measured by OCTA has been proved to be a sensitive and quantitative biomarker for monitoring cardiovascular risk.^26^, ^27^

In the current study, OCTA was used to explore the characteristics of retinal microvascular changes in both MHO and MetS patients and the impact of bariatric surgery on retinal microcirculation. The aim of our study is to elucidate the mechanism of cardiovascular complications caused by obesity and the mechanism by which bariatric surgery reduces cardiovascular risk.

## 2 MATERIALS AND METHODS

### 2.1 Subjects

Patients with obesity (including MetS and MHO) and age‐matched normal participants (controls) were enrolled in the Department of Ophthalmology at Huashan Hospital, Fudan University, between July 2018 and December 2020. All patients with obesity underwent laparoscopic sleeve gastrectomy in the Department of Metabolic Surgery at Huashan Hospital, and ophthalmic follow‐ups were performed before bariatric surgery and 6 months after surgery. This study was approved by the Huashan Hospital Institutional Review Board and conducted adhered to the tenets of the Declaration of Helsinki. Written informed consent was obtained from every participant prior to enrollment in the study.

The inclusion criteria were patients with obesity who were evaluated by Department of Metabolic Surgery for laparoscopic sleeve gastrectomy, with body mass index (BMI) ≥28 kg/m^2^ and with an age between 16 and 65 years. According to the Chinese Diabetes Society criteria,^28^ MetS was defined as having two or more of the following components: (1) fasting plasma glucose ≥6.1 mmol/L or 2‐h plasma glucose ≥7.8 mmol/L or self‐reported diabetes or taking antidiabetic medication; (2) systolic blood pressure (SBP) ≥130 mm Hg or diastolic blood pressure (DBP) ≥85 mm Hg or self‐reported hypertension or taking antihypertensive medication; (3) triglycerides (TG) ≥1.70 mmol/L; and (4) high‐density lipoprotein‐cholesterol (HDL‐c) <1.04 mmol/L. The waist circumference criterion was not used because of its collinearity with BMI. Participants who met fewer than two of the four criteria were considered MHO.

The exclusion criteria were as follows: (1) age >65 years; and (2) any coexisting retinal and ocular diseases, including diabetic retinopathy, choroidal neovascularization, glaucoma, optic neuritis, high refractive hyperopia or myopia (more than +6 or −6 diopters), opacities of the ocular media, and ophthalmic surgical treatment.

Control eyes were age‐matched healthy volunteers with a refractive error of less than six diopters and without any systemic, retinal, and ocular diseases, as described.^29^

All subjects underwent complete ophthalmic examinations, including best corrected visual acuity, noncontact tonometer intraocular pressure measurement, slit‐lamp biomicroscopy, fundus examination, and OCTA examination (Optovue Inc., Fremont, California, USA; soft‐ware version 2017.1.0.155).

### 2.2 Image acquisition and analysis

RTVue‐XR Avanti OCTA system (Optovue, Inc., Fremont, CA, USA) with split spectrum amplitude‐decorrelation angiography software was used to detect the microvascular network of study subjects. This device has an A‐scan rate of 70 kHz scans per second, using a light source centered on 840 nm. Each B‐scan in the OCT data frame consists of 304 A‐scans and is repeated twice for OCTA at the same scanning position. The whole data frame contains 304 B‐scans. Finally, 304 × 304 pixel OCTA was obtained.

OCTA examination was performed and read by the same experienced professional ophthalmic technician. The scanning procedure used was: 6 × 6 mm macular angiography (Angio Retina 6.0 mm). It was considered eligible for the current study that the OCTA images of the eyes kept reasonably still without significant movement or shadow artifacts and with a signal strength index score greater than Q6.

The macular OCTA images were analyzed using the built‐in software (Optovue Inc.; software version 2017.1.0.155). Vessel density was measured in four vascular layers, including superficial retinal capillary plexus (SCP), deep retinal capillary plexus (DCP), outer retina, and choriocapillaris plexus (CC). SCP is defined as the blood flow information between the inner boundary membrane and 10 microns above the lower edge of the inner plexus layer. DCP is defined as the blood flow information between 10 microns above the lower edge of the inner plexus layer to 10 microns below the outer plexus layer. The outer retina is defined as between 10 microns below the outer plexiform layer and 10 microns above Bruch's membrane. CC is defined as blood flow information between 10 microns above Bruch's membrane and 30 microns below Bruch's membrane. The accuracy of the automated segmentation was checked and segmentation errors were corrected manually.^25^ Meanwhile, foveal avascular zone (FAZ) area, FAZ perimeter, FAZ acircularity index (AI), and foveal density (FD) were also obtained by built‐in software.

### 2.3 Assessment of systemic data

Assessment of systemic data included standardized measurement of height, weight, BMI, SBP, DBP, and laboratory test. Laboratory tests were composed of blood routine, urine routine, liver function, renal function, lipid profile, blood glucose, blood insulin, and inflammatory factor profile. All blood and urine samples were collected at 6:00 am.

### 2.4 Statistical analysis

Statistical analysis was performed using IBM SPSS 23.0 software (Chicago, IL, USA). A Shapiro–Wilk test was used to test normal distribution for all continuous variables. Normally distributed variables were presented as mean ± SD, and variables from nonnormal distribution were presented as medians and interquartile ranges. One‐way analysis of variance (ANOVA) was used to compare the difference of macular retinal vessel density of the three groups of subjects. One‐way repeated measures ANOVA was used to compare the changes of macular retinal vessel density and systemic indexes before surgery and 6 months after surgery. Pearson correlation analysis and multiple linear regression analysis were used to study the correlation between the baseline or changes of macular retinal vessel density and systemic indexes in patients with obesity.

## 3 RESULTS

### 3.1 General characteristics

The general characteristics of enrolled subjects are presented in Table 1. A total of 84 patients with MetS, 52 patients with MHO, and 52 normal‐weight, age‐matched controls were enrolled in the present study.

**TABLE 1:** Characteristics of the enrolled groups.

Characteristic	MetS	MHO	Controls	*p* value
Number of patients enrolled	84	52	52	
Age, years	29.5 ± 6.5	30.6 ± 8.8	30.5 ± 7.2	.601^a^
Gender, male/female	30/54	16/36	17/35	.829^b^
BMI, kg/m^2^	39.5 ± 7.0	37.5 ± 6.0	21.4 ± 3.0	<.001^a^, ***
Hypertension, *n*(%)	16(19.05%)	1(1.89%)	0(0%)	<.001^b^, ***
Diabetes, *n* (%)	23(27.38%)	0(0%)	0(0%)	<.001^b^, ***
SBP, mm Hg	135 ± 13	122 ± 15	116 ± 7	<.001^a^, ***
DBP, mm Hg	88 ± 12	79 ± 11	74 ± 7	<.001^a^, ***
Glucose, mmol/L	6.98 ± 2.87	5.25 ± 0.50	4.98 ± 0.39	<.001^a^, ***
Total cholesterol, mmol/L	4.91 ± 0.98	4.81 ± 0.73	4.64 ± 0.84	.483^a^
Triglycerides, mmol/L	2.11 ± 1.28	1.48 ± 0.81	1.27 ± 1.41	.002^a^, **
LDL‐cholesterol, mmol/L	3.22 ± 0.87	3.22 ± 0.69	2.27 ± 0.83	.004^a^, **
HDL‐cholesterol, mmol/L	1.00 ± 0.22	1.09 ± 0.17	1.53 ± 0.28	<.001^a^, ***
Insulin, μIU/mL	37.94 ± 25.22	27.80 ± 14.46		.012^c^, *
HbA_1c_, %	6.91 ± 1.56	5.71 ± 0.40		<.001^c^, ***
hsCRP, mg/L	6.16 ± 3.10	4.43 ± 3.27		.003^c^, **
Course, years	12 ± 8	11 ± 8		.531^a^

Abbreviations: BMI, body mass index; DBP, diastolic blood pressure; HbA_1c_, glycosylated hemoglobin; HDL, high‐density lipoprotein; hsCRP, high‐sensitivity C‐reactive protein; LDL, low‐density lipoprotein; MetS, metabolic syndrome; MHO, metabolically healthy obesity; SBP, systolic blood pressure.

^a^ One‐way analysis of variance.

^b^ Chi‐square test.

^c^ *t* test.

* *p* < .05;

** *p* < .01;

*** *p* < .001.

We found no significant difference among the three groups with regard to age (*p* = .601) and gender distribution (*p* = .829). There were significant differences in BMI among the three groups (*p* < .05). Furthermore, a pair‐based comparison showed that the BMI of MetS and MHO group were significantly higher than that of controls (*p* < .05), whereas there was no statistically significant difference in BMI between the MetS and the MHO group (*p* = .103). Sixteen patients (19.05%) in the MetS group and 1 patient (1.89%) in the MHO group had hypertension. Twenty‐three patients (27.38%) in the MetS had diabetes. There were significant differences in SBP and DBP among the three groups (*p* < .05), and pair‐based comparison showed that the blood pressure of the MetS group was significantly higher than that of the MHO group and controls (*p* < .05). Also, there were significant differences in fasting plasma glucose among the three groups (*p* < .05), and pair‐based comparison showed that the blood glucose of the MetS group were significantly higher than that of the MHO group and controls (*p* < .05). There were significant differences in TG, low‐density lipoprotein (LDL), and HDL among the three groups (*p* < .05). Pair‐based comparison showed that the TG of the MetS group were significantly higher than that of the MHO group and controls (*p* < .05), and the LDL of the MetS and MHO groups were significantly higher than that of controls (*p* < .05). For HDL parameters, the HDL of the MetS group was significantly lower than that of the MHO group, and the HDL of the MHO group was significantly lower than that of control group. There was no statistically significant difference in total cholesterol among the three groups (*p* = .483).

In addition, insulin, glycosylated hemoglobin (HbA_1c_), and high‐sensitivity C‐reactive protein (hsCRP) in the MetS group were significantly higher than those in the MHO group (for each *p* < .05). There was no statistically significant difference in disease course between the MetS and the MHO groups.

### 3.2 Baseline OCTA outcome

Eighty‐four eyes (84 patients) with MetS, 52 eyes (52 patients) with MHO, and 52 control eyes (52 controls) were included in the baseline study. All enrolled subjects underwent OCTA examination before bariatric surgery. The results of comparison of macular retinal vessel density among groups were shown in Table 2.

**TABLE 2:** Differences in optical coherence tomography angiography measurements among MetS, MHO, and control eyes.

Parameter	MetS	MHO	Controls	*p* value
Number of eyes	84	52	52	
SCP vessel density, %				
Whole image	50.58 ± 2.99	51.38 ± 2.76	51.65 ± 2.15	.059
Fovea	19.91 ± 6.11	19.21 ± 6.97	22.49 ± 7.09	.028*
Parafovea	52.61 ± 4.63	53.56 ± 3.36	53.68 ± 2.73	.203
Perifovea	51.51 ± 2.95	52.36 ± 2.81	52.53 ± 2.29	.071
DCP vessel density, %				
Whole image	51.60 ± 5.59	52.53 ± 5.84	54.20 ± 4.66	.028*
Fovea	36.64 ± 6.95	35.33 ± 7.82	39.14 ± 6.09	.019*
Parafovea	56.24 ± 3.99	56.84 ± 3.82	57.65 ± 3.72	.043*
Perifovea	52.59 ± 6.19	53.72 ± 6.29	55.58 ± 5.10	.019*
FAZ area, mm^2^	0.29 ± 0.10	0.31 ± 0.11	0.27 ± 0.08	.093
FAZ perimeter, mm	2.04 ± 0.39	2.12 ± 0.36	1.95 ± 0.39	.093
FAZ AI	1.10 ± 0.04	1.09 ± 0.02	1.08 ± 0.12	.173
FD, %	53.88 ± 4.53	55.17 ± 3.67	54.56 ± 8.55	.443

Abbreviations: AI, acircularity index; DCP, deep capillary plexus; FAZ, foveal avascular zone; FD, foveal density; MetS, metabolic syndrome; MHO, metabolically healthy obesity; SCP, superficial capillary plexus.

Bold face indicates statistically significant *p* value.

**FIGURE 1:**
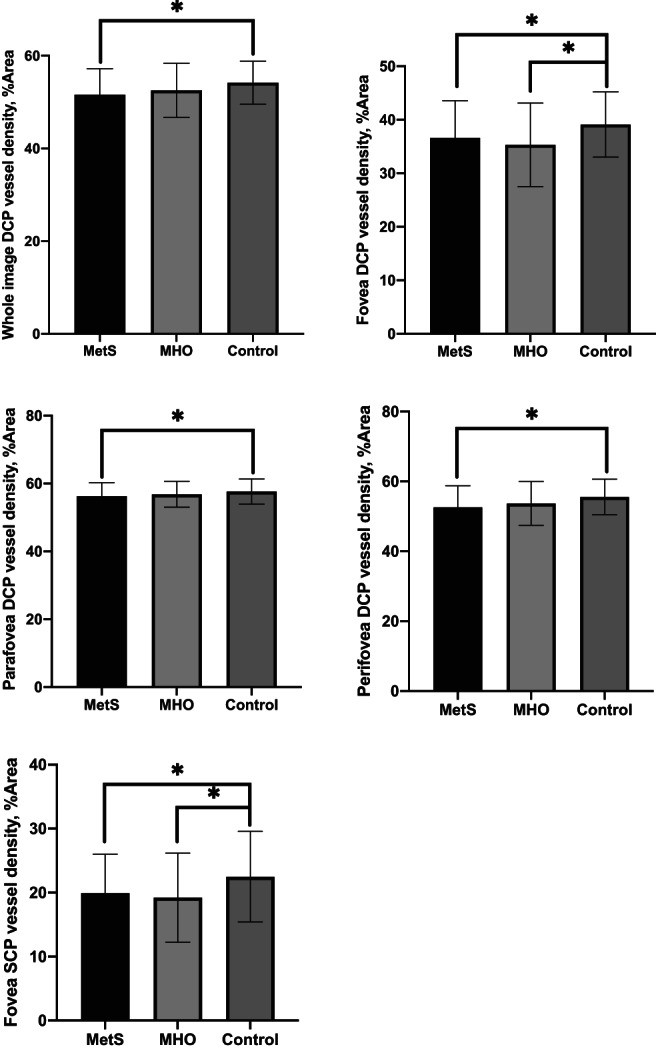
Retinal vessel density of MetS, MHO, and control eyes. Pair‐based comparison demonstrated that the whole image DCP, fovea DCP, parafovea DCP, perifovea DCP, and fovea SCP vessel densities were significantly lower in the MetS group, compared with the control group (for each *p* < .05). Fovea SCP and DCP vessel densities were significantly lower in MHO group compared to controls. *p < .05. DCP, deep capillary plexus; MetS, metabolic syndrome; MHO, metabolically healthy obesity; SCP, superficial capillary plexus.

For macular retinal vessel density, there were significant differences in fovea SCP, average DCP, fovea DCP, parafovea DCP, and perifovea DCP vessel densities among the three groups (for each *p* < .05). However, no significant difference was found in the average SCP (*p* = .059), parafovea SCP (*p* = .203), and perifovea SCP (*p* = .071) vessel densities among groups. As shown in Figure 1, a pair‐based comparison demonstrated that the fovea SCP, average DCP, fovea DCP, parafovea DCP, and perifovea DCP vessel densities were significantly lower in the MetS group, compared with the control group (19.91 ± 6.11% vs. 22.49 ± 7.09%, 51.60 ± 5.59% vs. 54.20 ± 4.66%, 36.64 ± 6.95% vs. 39.14 ± 6.09%, 56.24 ± 3.99% vs. 57.65 ± 3.72% and 52.59 ± 6.19% vs. 55.58 ± 5.10%, respectively, all *p* < .05). Meanwhile, most macular vessel densities in the MetS group showed a decreasing trend compared with the MHO group, although the difference was not statistically significant (*p* > .05). Fovea SCP and fovea DCP vessel densities were significantly lower in the MHO group compared to controls (19.21 ± 6.97% vs. 22.49 ± 7.09% and 35.33 ± 7.82% vs. 39.14 ± 6.09%, respectively, all *p* < .05). However, there was no significant difference in other macular vessel densities between the MHO and control groups.

For FAZ parameters, there were no significant differences in FAZ area, FAZ perimeter, FAZ AI, and FD among the three groups.

**FIGURE 2:**
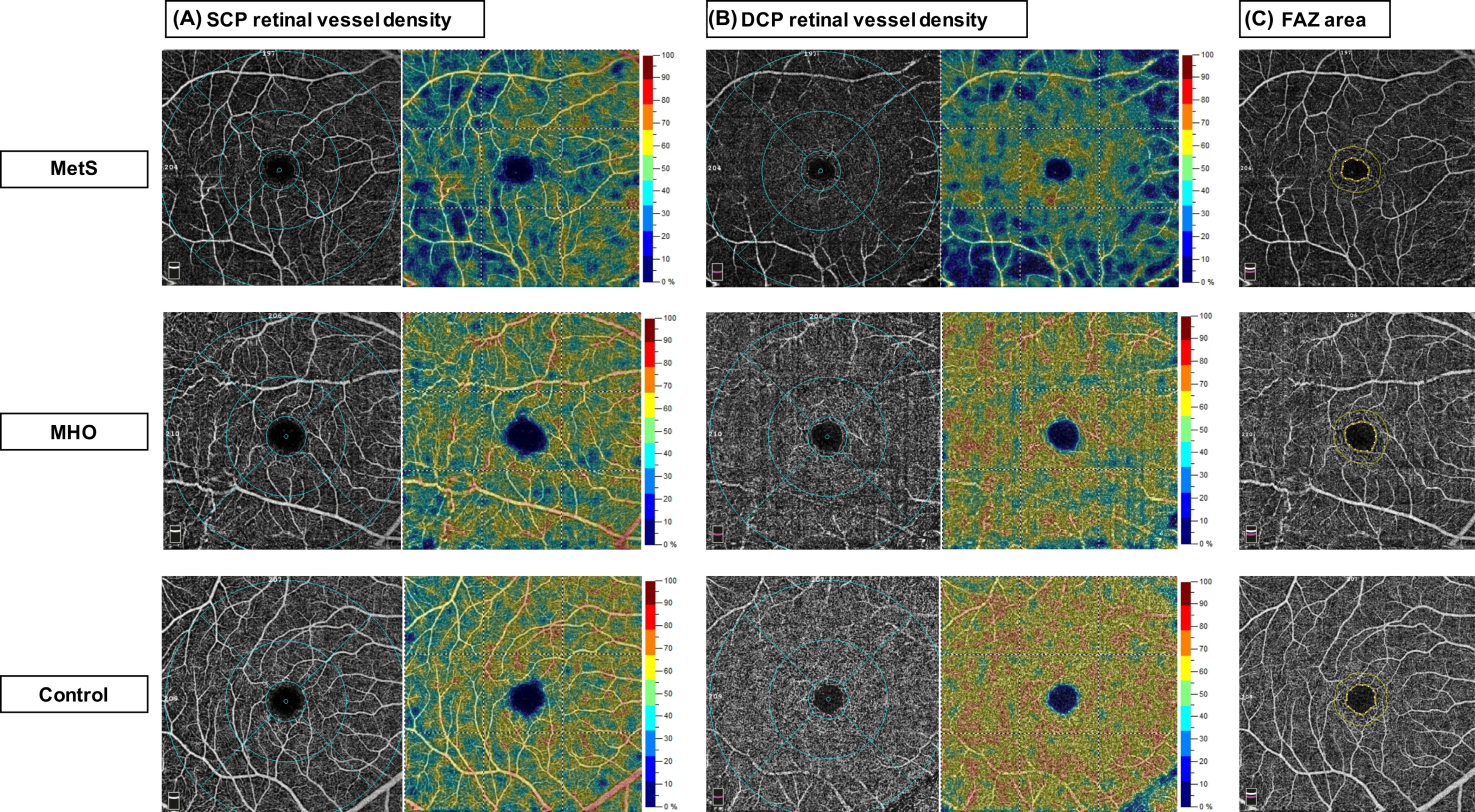
Representative OCTA images for comparisons of retinal vessel density and foveal avascular zone measurements among MetS, MHO, and control eyes. (A) OCTA images of SCP retinal vessel density. (B) OCTA images of DCP retinal vessel density. (C) OCTA images of FAZ area. DCP, deep capillary plexus; FAZ, foveal avascular zone; MetS, metabolic syndrome; MHO, metabolically healthy obesity; SCP, superficial capillary plexus.

Representative cases of OCTA images for comparisons of retinal vessel density and FAZ measurements among MetS, MHO, and control eyes are shown in Figure 2.

### 3.3 Microvascular changes after bariatric surgery

Nineteen eyes from 19 patients with obesity (including 9 males and 10 females) were included in the longitudinal study. The mean age was 29.7 ± 9.9 years. Among them, eight patients had MHO and 11 patients had MetS. Follow‐up was performed 6 months after surgery. The comparison of anthropometric and microvascular parameters in patients with obesity at baseline and 6 months after surgery was shown in Table 3.

**TABLE 3:** Anthropometric and microvascular parameters at baseline and 6 months after bariatric surgery.

Parameter	Baseline	6 m‐post	*p* value
Number of patients (eyes)	19 (19)	19 (19)	
BMI, kg/m^2^	39.1 ± 6.8	27.7 ± 4.8	<.001***
SBP, mm Hg	128.5 ± 16.2	122.7 ± 14.6	.033*
DBP, mm Hg	81.1 ± 8.9	76.6 ± 9.0	.012*
Insulin, μIU/mL	39.33 ± 24.45	8.58 ± 6.67	<.001***
HbA_1c_, %	6.40 ± 1.64	5.46 ± 0.43	.002**
Triglycerides, mmol/L	1.56 ± 0.65	0.87 ± 0.29	<.001***
hsCRP, mg/L	5.31 ± 3.24	2.53 ± 3.11	<.001***
SCP vessel density, %			
Whole image	50.68 ± 3.50	51.60 ± 3.21	.178
Fovea	23.08 ± 5.34	23.76 ± 5.01	.536
Parafovea	52.97 ± 3.67	54.21 ± 3.39	.042*
Perifovea	51.40 ± 3.91	52.23 ± 3.32	.272
DCP vessel density, %			
Whole image	50.95 ± 6.01	54.43 ± 6.65	.041*
Fovea	40.29 ± 4.56	40.68 ± 4.08	.426
Parafovea	55.54 ± 3.79	58.29 ± 4.39	.017*
Perifovea	51.82 ± 6.67	55.76 ± 7.21	.032*
FAZ area, mm^2^	0.25 ± 0.06	0.25 ± 0.06	.420
FAZ perimeter, mm	1.94 ± 0.22	1.92 ± 0.22	.134
FAZ AI	1.10 ± 0.02	1.09 ± 0.02	.138
FD, %	53.97 ± 3.76	55.55 ± 3.21	.103

Abbreviations: AI = acircularity index; BMI = body mass index; DBP = diastolic blood pressure; DCP = deep capillary plexus; FAZ = foveal avascular zone; FD = foveal density; HbA_1c_ = glycosylated hemoglobin; hsCRP = high‐sensitivity C‐reactive protein; SBP = systolic blood pressure; SCP = superficial capillary plexus.

Bold face indicates statistically significant *p* value.

**FIGURE 3:**
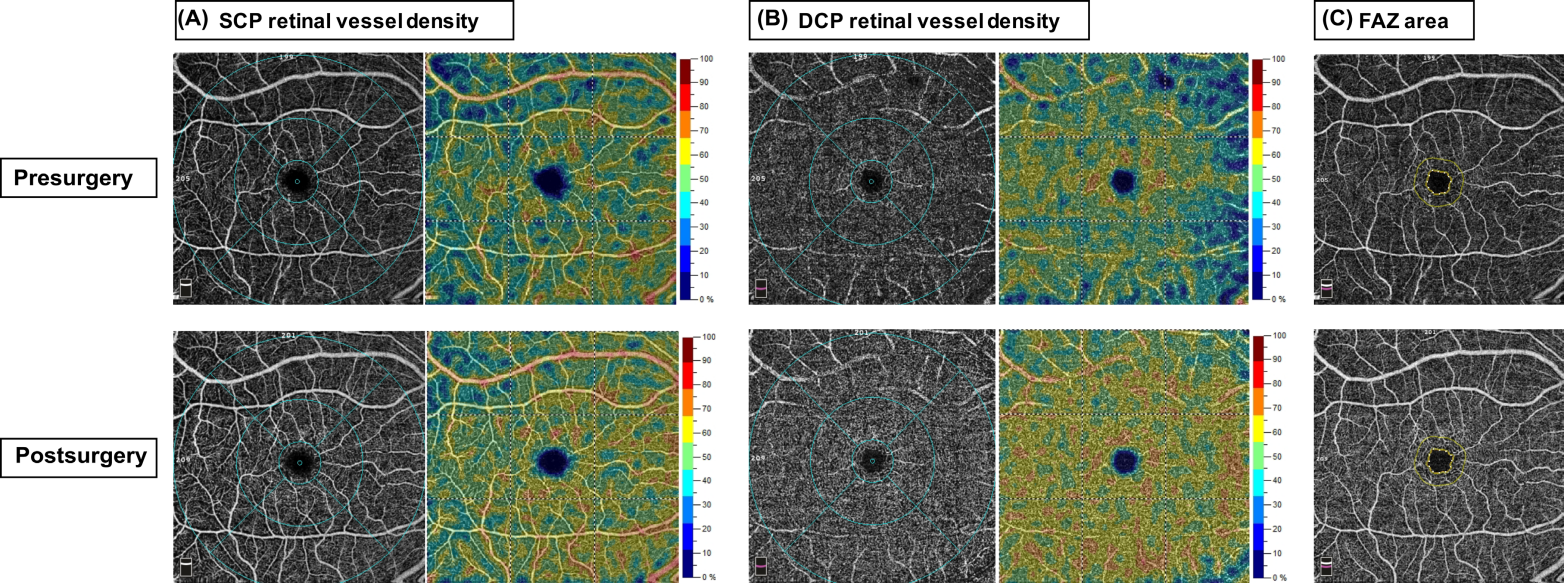
Effects of bariatric surgery on retinal vessel density and foveal avascular zone of representative patients with obesity. DCP vessel density significantly improved at 6 months post bariatric surgery. (A) OCTA images of SCP retinal vessel density. (B) OCTA images of DCP retinal vessel density. (C) OCTA images of FAZ area. DCP, deep capillary plexus; FAZ, foveal avascular zone; MetS, metabolic syndrome; MHO, metabolically healthy obesity; SCP, superficial capillary plexus.

Compared with baseline, the parafovea SCP, average DCP, parafovea DCP, and perifovea DCP vessel densities were significantly increased in patients with obesity 6 months after surgery (54.21 ± 3.39% vs. 52.97 ± 3.67%, 54.43 ± 6.65% vs. 50.95 ± 6.01%, 58.29 ± 4.39% vs. 55.54 ± 3.79% and 55.76 ± 7.21% vs. 51.82 ± 6.67%, respectively, all *p* < .05). At 6 months after surgery, the average SCP, fovea SCP, perifovea SCP, and fovea DCP vessel densities were slightly higher than baseline without statistically significant (*p* > .05). For FAZ parameters, there was no significant change in FAZ area, FAZ perimeter, AI, and FD of patients with obesity after surgery. Representative cases of OCTA images for comparisons of retinal vessel density and FAZ measurements before and after bariatric surgery are shown in Figure 3.

In terms of systemic and metabolic indicators, BMI decreased significantly in patients with obesity at 6 months after surgery compared with baseline (*p* < .05). Meanwhile, SBP, DBP, plasma fasting insulin, HbA_1c_, TG, and hsCRP were significantly decreased 6 months after surgery (for each *p* < .05).

### 3.4 Correlation and regression analysis

Correlation analysis between baseline systemic data and OCTA parameters was shown in Table 4. In the MHO group (Table 4), average SCP vessel density was negatively correlated with BMI (*r* = −0.399, *p* = .002), hsCRP (*r* = −0.367, *p* = .007), and plasma fasting insulin (*r* = −0.291, *p* = .036) and was positively correlated with TG (*r* = 0.302, *p* = .029). Meanwhile, average DCP vessel density was negatively correlated with BMI (*r* = −0.314, *p* = .018) and hsCRP (*r* = −0.351, *p* = .011). In the MetS group (Table 4), average SCP vessel density was negatively correlated with BMI (*r* = −0.255, *p* = .022) and plasma fasting insulin (*r* = −0.235, *p* = .037), and average DCP vessel density was negatively correlated with BMI.

**TABLE 4:** Correlation analysis between retinal vessel density and anthropometric variation in MetS and MHO patients.

Variable	Correlation coefficient	*p* value
MHO group		
Average SCP vessel density		
BMI	−0.399	.002**
hsCRP	−0.367	.007**
Fasting insulin	−0.291	.036*
Triglycerides	0.302	.029*
Average DCP vessel density		
BMI	−0.314	.018*
hsCRP	−0.351	.011*
MetS group		
Average SCP vessel density		
BMI	−0.255	.022*
Fasting insulin	−0.235	.037*
Average DCP vessel density		
BMI	−0.248	.028*

Abbreviations: BMI, body mass index; DCP, deep capillary plexus; hsCRP, high‐sensitivity C‐reactive protein; MetS, metabolic syndrome; MHO, metabolically healthy obesity; SCP, superficial capillary plexus.

Furthermore, multivariable regression analysis adjusted for age and sex was performed to evaluate the independent association between baseline systemic data and macular vessel densities (see Table S1 in Appendix S1). Our results showed that BMI (*p* = .01) and hsCRP (*p* = .019) were independent predictors of average SCP vessel density at baseline in the MHO group. Similarly, BMI (*p* = .045) and hsCRP (*p* = .022) could independently predict average DCP vessel density in the MHO group. For the MetS group, no independent predictor of macular vessel densities was found in multivariable regression analysis.

Also, multivariable regression analysis was performed to evaluate the independent association between baseline systemic data and changes of macular vessel densities after surgery (Table 5). Baseline DBP (*p* = .037) and plasma fasting insulin (*p* = .002) were independent predictors of average SCP vessel density changes 6 months after surgery. Over the 6‐month follow‐up, for every 1 mm Hg increase of baseline DBP, postoperative SCP vessel density increased by 0.205%. For every 1 mU/L increase of baseline fasting plasma insulin, postoperative SCP vessel density decreased by 0.063%. Baseline SBP (*p* = .023) and plasma fasting insulin (*p* = .009) were independent predictors of changes in DCP vessel density 6 months after surgery. Over the 6‐month follow‐up, for every 1 mm Hg increase of baseline SBP, postoperative DCP vessel density increased by 0.239%. For every 1 mU/L increase of baseline plasma fasting insulin, the postoperative DCP vessel density decreased by 0.1%.

**TABLE 5:** Multivariable regression analysis between change in retinal vessel density and baseline anthropometric variation.

Factor	Beta value	*R* ^2^	Adjusted *R* ^2^	*p* value
SCP vessel density				
DBP	0.205	0.494	0.392	.037*
Fasting insulin	−0.063	0.646	0.575	.002**
DCP vessel density				
SBP	0.239	0.398	0.277	.023*
Fasting insulin	−0.100	0.466	0.359	.009**

Abbreviations: DBP, diastolic blood pressure; DCP, deep capillary plexus; SBP, systolic blood pressure; SCP, superficial capillary plexus.

## 4 DISCUSSION

In our present study, significant reduction of retinal vessel densities was detected by OCTA in the MetS group rather than the MHO group, compared to healthy controls. Our results identified differences in microvascular phenotypes by metabolic status, underscoring heterogeneity in vascular physiology of adiposity and potential activation of distinct pathological pathways in clinical obesity subgroups. In addition, our follow‐up investigation indicated that bariatric surgery improved microvascular status of patients with obesity, and baseline blood pressure as well as fasting insulin were independent predictors of retinal vascular benefit 6 months after surgery. This finding may have clinical implications with regard to treatment prioritization of those patients who gain more from bariatric surgery.

Overweight and obesity are associated with an increased risk of microvascular dysfunction and related cardiometabolic disease.^3^, ^4^, ^5^ Retinal microvasculature measures have been proved to be surrogate markers of microvascular disease and predictors of future cardiometabolic disease.^10^, ^11^ Previous studies identified the association between BMI and retinal vascular caliber.^12^, ^13^ Boillot et al conducted a systematic review and meta‐analysis of over 44 000 individuals and found that higher BMI was associated with narrower retinal arteriolar and wider venular calibers, independent of other cardiovascular risk factors.^12^ Besides, wider retinal venular diameters may predict development of obesity.^13^ However, these previous studies used static and manually measured retinal vascular parameters, which could not be widely used in clinical practice.

In the present study, we used OCTA to dynamically assess retinal perfusion. Results of our study confirmed that retinal vessel densities were significantly reduced in patients with obesity, in line with previous findings. As a noninvasive and OCT‐based technique, OCTA may provides robust microvasculature quantification in patients with obesity, with nearly no ocular or systemic side effect.^23^, ^24^, ^25^, ^26^, ^27^ In addition to OCTA, several retinal imaging techniques could also provide in vivo information of the microvascular health, such as retinal fundus photography and dye‐based ocular angiography. Fundus photography is a static retinal imaging technique that could provide only structural vascular information.^12^ Dye‐based ocular angiography is an invasive procedure with an associated risk of complications or adverse reactions such as allergic response and discoloration of the skin.^30^ Taken together, we suggest that OCTA may be a reliable tool to detect retinal microvascular changes and access the systemic microcirculation status in obesity.

To the best of our knowledge, this is the first study to differentiate the retinal microvascular characteristic in obese population with the MetS versus MHO population. Previous studies on obesity and microvascular dysfunction have focused on the relationship between BMI and microcirculation,^10^, ^11^, ^12^, ^13^ and the role of metabolic status in the microvascular changes associated with obesity remains unclear. In the present study, we identified different microvascular phenotypes of patients with obesity using OCTA, showing that MetS patients exhibited more severe microvascular dysfunction than those with MHO.

As an active endocrine and metabolic organ, adipose tissue interacts and undergoes cross‐talk with multiple systems.^31^, ^32^ Obesity could induce systemic perturbations to organismal metabolism, leading to hyperglycemia, hypertension, dyslipidemia, insulin resistance, and baseline changes in inflammation. Yet studies have shown that about 31.7% of patients with obesity are not accompanied by obvious endocrine disorders and metabolic disorders,^33^ therefore emphasis has been increasingly placed on the metabolic status of patients with obesity, rather than BMI. Our current study suggests that metabolic status may play an important role in obesity‐related microvascular dysfunction, which unveils the mechanism underlying different cardiometabolic outcomes between MetS and MHO. In addition, multivariable regression analysis showed that microvascular densities decreased with the increase of BMI or systemic inflammation level in the MHO group, which is consistent with the results of previous large‐sample population studies. We conclude that the proinflammatory state associated with obesity may be an early pathological mechanism of microcirculatory damage in obesity, prior to other metabolic factors.

In the present study, we also aim to investigate the beneficial impact of bariatric surgery on microcirculation. Our results demonstrated that retinal vessel densities in patients with obesity are significantly improved 6 months after bariatric surgery, mainly manifested by the increase of DCP vessel densities. Two previous studies have assessed the effects of bariatric surgery directly on the retinal microvasculature. Viljanen et al showed that over 6 months, bariatric surgery improved the structure of the retinal microvasculature, leading to a reduction in retinal arteriolar narrowing and venular widening.^34^ Streese et al followed a group for 6 weeks post bariatric surgery and demonstrated that bariatric surgery primarily affects microvascular phenotype rather than large artery stiffness.^22^ However, only static retinal imaging was employed in prior studies; meanwhile, populations enrolled in these studies had small sample size and unrepresentative gender distribution. Our study using OCTA confirmed previous findings that bariatric surgery improves the microcirculation of patients with obesity. Notably, some of these changes of vessel densities might be attributed to intrasession and between‐visit variability of the device and might not be clinically significant.^35^ Future prospective studies with a larger sample size and a follow‐up of control group are warranted to verify this point. Collectively, OCTA seems to be a feasible tool to monitor microvascular health and cardiometabolic risk after bariatric surgery.

The underlying mechanisms for the beneficial effects of bariatric surgery on retinal microvasculature remain unclear. The present study showed that baseline blood pressure and plasma fasting insulin are independent predictors of changes in retinal vessel densities 6 months after surgery. Our finding provide a potential mechanism for microvascular benefit from bariatric surgery, suggesting that baseline blood pressure and insulin status may be key determinants. Meanwhile, our finding may have clinical implications with regard to treatment prioritization of those patients who gain more from bariatric surgery.

First, significant reduction in blood pressure after weight loss may be an important factor leading to microvascular improvements.^36^, ^37^ Hughes et al prospectively evaluated the effect of lowered blood pressure on retinal microcirculation and found that retinal arterioles widened as well as retinal arteriolar density improved after 12 months of antihypertensive treatment.^37^ This is consistent with our finding, implying that bariatric surgery may improve microcirculation status through significant hypotensive effect, thus obese individuals with preoperative hypertension may gain better vascular benefit.

Second, baseline insulin level may also play a role in the changes of microcirculation caused by bariatric surgery. Increased insulin sensitivity after bariatric surgery may involve complex beneficial mechanisms, including regulation of adipose tissue phenotypes, reduction of adipose‐derived inflammatory cytokines, fatty acid mobilization, and hormonal shifts.^38^, ^39^ In the present study, bariatric surgery significantly improved insulin resistance and microcirculation disorders in patients with obesity; meanwhile, baseline insulin level was negatively correlated with the changes of retinal vessel densities 6 months after surgery. Our results suggested that obese individuals with lower preoperative insulin level have greater microvascular benefits short‐term after bariatric surgery. However, this result may be controversial. Bigornia et al previously reported that patients with high‐risk hyperinsulinemia most likely benefited from weight reduction.^40^ Given that the study of Bigornia et al enrolled up to 85% female patients and the average baseline insulin level of their cohort was lower than that of our study, further studies with a large sample size are needed to clarify the impact of baseline insulin level on the prognosis of microcirculation in patients with obesity after weight loss.

Our study has several possible limitations. First, we did not examine the cardiovascular outcome data. Nevertheless, our end points of retinal vascular densities as surrogates of cardiovascular outcome are warranted. Several studies indicate that noninvasive retinal microvasculature measures can serve as biomarkers for future cardiometabolic diseases.^10^, ^11^ Second, there was a relatively large lost to follow‐up rate in the present study, which may lead to bias of our results. A proportion of the lost to follow‐up patients were referred from other provinces to our metabolic surgery center for bariatric surgery and received postoperative follow‐up in their local hospitals. Another part of these patients were lost due to COVID‐19 lockdown. However, a comparison of baseline characteristics between the follow‐up group and the lost to follow‐up group showed no significant difference (see Table S2). Additional prospective studies with large sample sizes are needed to confirm our conclusions. Third, the presence of diabetes (27.38%) and hypertension (19.05%) may be confounders in microvascular dysfunction of MetS group in our study. However, previous studies have reported that microvascular dysfunction was present in MetS patients without diabetes and hypertension.^41^, ^42^ Furthermore, there were no significant alterations to our results when diabetes and hypertension history or status were accounted for (see Table S3). Fourth, owing to the relatively small sample size, we are not able compare the trend sufficiently in vessel density changes between MetS and MHO groups. Future study with a larger sample size is warranted to clarify this important issue. Lastly, we did not exclude the possible confounding effects of diet, exercise, or other lifestyle treatments as well as medication. Future investigation is warranted to eliminate the influence these confounding factors.

In conclusion, our study found that retinal microvascular impairment occurred in MetS patients rather than MHO patients and bariatric surgery could significantly improve the microcirculation status of patients with obesity. Our results suggest that the advanced noninvasive retinal microvasculature measure, OCTA, may be a reliable method to study the onset and progression of microvascular complications associated with obesity before and after bariatric surgery. Baseline blood pressure and insulin status may be key determinants for vascular benefit from bariatric surgery.

## CONFLICT OF INTEREST STATEMENT

All the authors declare no conflicts of interest.

## Supporting information


**Table S1.** Multivariable regression analysis between retinal vessel density and anthropometric variation in metabolically healthy obesity patients
**Table S2.** Comparison of baseline characteristics between the follow‐up and lost to follow‐up obesity patients.
**Table S3.** Differences in microvascular measurements between metabolic syndrome without hypertension or diabetes and control eyes.
