# Supplementary material for: Sleeve gastrectomy improved microvascular phenotypes from obesity cohort, detected with optical coherence tomography angiography
Source: J Diabetes. 2023 Mar 5;15(4):313–24. doi: 10.1111/1753-0407.13374 (PMC10101840; doi:10.1111/1753-0407.13374)
Supplement: Supplementary file 1 — Table S1. Multivariable regression analysis between retinal vessel density and anthropometric variation in metabolically healthy obesity patients Table S2. Comparison of baseline characteristics between the follow‐up and lost to follow‐up obesity patients. Table S3. Differences in microvascular measurements between metabolic syndrome without hypertension or diabetes and control eyes. [file JDB-15-313-s001.docx]

**Supplementary Appendix**

**Table 1.** Multivariable regression analysis between retinal vessel density and anthropometric variation in MHO patients

| Factor | Beta value | R squared | Adjusted R squared | P value |
| --- | --- | --- | --- | --- |
| SCP vessel density | | | | |
| BMI | -0.181 | 0.201 | 0.155 | 0.010* |
| hsCRP | -0.318 | 0.189 | 0.138 | 0.019* |
| DCP vessel density | | | | |
| BMI | -0.293 | 0.121 | 0.071 | 0.045* |
| hsCRP | -0.628 | 0.148 | 0.095 | 0.022* |

Abbreviations: BMI = body mass index; hsCRP = high-sensitivity C-reactive protein; SCP = superficial capillary plexus; DCP = deep capillary plexus.

**Table 2.** Comparison of baseline characteristics between the follow-up and lost to follow-up obesity patients

|  | Follow-up patients | Lost to follow-up patients | P value |
| --- | --- | --- | --- |
| Number of patients | 19 | 117 |  |
| Age, years | 29.7±9.9 | 29.6±7.2 | 0.934 |
| Gender, male/female | 9/10 | 37/80 | 0.170 |
| BMI, kg/m^2^ | 38.9±6.8 | 38.6±6.7 | 0.853 |
| SBP, mmHg | 128±16 | 130±15 | 0.541 |
| DBP, mmHg | 81±9 | 85±13 | 0.273 |
| Total cholesterol, mmol/L | 4.88±1.25 | 4.82±0.84 | 0.806 |
| Triglycerides, mmol/L | 1.56±0.66 | 1.88±1.20 | 0.252 |
| LDL-cholesterol, mmol/L | 3.25±1.13 | 3.18±0.74 | 0.806 |
| HDL-cholesterol, mmol/L | 1.04±0.23 | 1.03±0.20 | 0.840 |
| Glucose, mmol/L | 6.39±1.66 | 6.30±2.51 | 0.878 |
| Insulin, μIU/mL | 47.15±41.77 | 33.30±21.62 | 0.173 |
| HbA_1c_, % | 6.51±1.28 | 6.44±1.38 | 0.831 |
| hsCRP, mg/L | 5.31±3.28 | 5.54±3.32 | 0.772 |
| Course, years | 15±9 | 11±8 | 0.050^c^ |

Abbreviations: MetS = metabolic syndrome; MHO = metabolically healthy obesity; BMI = body mass index; SBP = systolic blood pressure; DBP = diastolic blood pressure; LDL = low-density lipoprotein; HDL = high-density lipoprotein; HbA_1c_ = glycosylated hemoglobin; hsCRP = high-sensitivity C-reactive protein.

**Table 3.** Differences in microvascular measurements between MetS without hypertension or diabetes and control eyes

|  | MetS-NHBP | MetS-NDM | Controls | P_1_ value | P_2_ value |
| --- | --- | --- | --- | --- | --- |
| Number, eyes | 68 | 61 | 52 |  |  |
| SCP vessel density, %  Whole image  Fovea  Parafovea  Perifovea | 50.75±3.11  19.86±5.91  52.91±4.85  51.63±3.06 | 50.76±2.71  19.01±6.41  52.98±4.22  51.75±2.60 | 51.65±2.15  22.49±7.09  53.68±2.73  52.53±2.29 | 0.065  **0.030***  0.275  0.068 | 0.060  **0.008****  0.288  0.095 |
| DCP vessel density, %  Whole image  Fovea  Parafovea  Perifovea | 51.44±5.60  36.67±7.06  56.28±3.95  52.41±6.19 | 52.31±5.17  35.89±7.23  56.85±3.48  53.41±5.62 | 54.20±4.66  39.14±6.09  57.65±3.72  55.58±5.10 | **0.005****  **0.048***  0.058  **0.003**** | **0.047***  **0.013***  0.247  **0.036*** |

Abbreviations: MetS = metabolic syndrome; MetS-NHBP = metabolic syndrome without high blood pressure; MetS-NDM = metabolic syndrome without diabetes mellitus; SCP = superficial capillary plexus; DCP = deep capillary plexus; P_1_ value = P value of MetS-NHBP vs controls; P_2_ value = P value of MetS-NDM vs controls.
